# The Four-Herb Chinese Medicine ANBP Enhances Wound Healing and Inhibits Scar Formation via Bidirectional Regulation of Transformation Growth Factor Pathway

**DOI:** 10.1371/journal.pone.0112274

**Published:** 2014-12-09

**Authors:** Qian Hou, Wen-Jun He, Hao-Jie Hao, Qing-Wang Han, Li Chen, Liang Dong, Jie-Jie Liu, Xiang Li, Ya-Jing Zhang, Ying-Zhi Ma, Wei-Dong Han, Xiao-Bing Fu

**Affiliations:** 1 Institute of Basic Medicine, Chinese PLA General Hospital, Beijing, P. R. China; 2 Department of Internal Medicine, Chinese PLA 148th Hospital, Zibo, P. R. China; 3 Wound Healing and Cell Biology Laboratory, the First Affiliated Hospital, Chinese PLA General Hospital, Beijing, P. R. China; Institut national de la santé et de la recherche médicale - Institut Cochin, France

## Abstract

The four-herb Chinese medicine ANBP is a pulverized mixture of four herbs including *Agrimonia Eupatoria* (A), *Nelumbo Nucifera Gaertn* (N), *Boswellia Carteri* (B) and *Pollen Typhae Angustifoliae* (P). The combination of the four herbs was first described in Chinese canonical medicine about 2000 years ago for treatment of various trauma disorders, such as hemostasis, antiinflammatory, analgesia, and wound healing, etc. However, the precise mechanisms of ANBP are still unclear. In our study, using rabbit ear hypertrophic scar models of full-thickness skin defect, we showed that local ANBP treatment not only significantly enhanced wound healing by relieving inflammation, increasing formation of granulation tissue and accelerating re-epithelialization, but also reduced scar formation by decreasing collagen production, protuberant height and volume of scars, and increasing collagen maturity. We demonstrated that these effects of ANBP are associated with transforming growth factor (TGF)-β1-mediated signalling pathways through Smad-dependent pathways. ANBP treatment significantly increased expression of TGF-β1 and Smad2/3 mRNA at the early stage of wound healing, and led to markedly decrease expression of TGF-β1 and Smad2/3 compared with the control group after 14 days post-wounding. Taken together, our results defined a bidirectional regulation role of ANBP for TGF-β1/Smad pathway in promoting wound healing and alleviating scar formation, which may be an effective therapy for human wounds at the earliest stage.

## Introduction

Wound healing is a dynamic and complex process controlled by many factors. Once the protective barriers of epidermis and dermis are broken, the physiologic process of wound healing is immediately carried on. Wound healing involves distinct overlapping phases of coagulation, inflammation, proliferation and tissue remodeling [1]–[3]. During this process, a set of complex biochemical events takes place in a closely orchestrated cascade to repair the damage. Errors in wound healing can lead to delayed healing or formation of hypertrophic scars [4]. The goal for wound treatment is fast and scarless healing. However, fast healing and scar formation is a pair of contradiction in clinical treatment. There is no wide accepted regimen to both improve wound healing and reduce scar formation. The current treatments [5], which include chemical drugs, pressure therapy, laser therapy, radiation and surgical operation, could not achieve the satisfied results.

With rich resource, low cost and few side effects, natural Chinese herb medicines composed of multiple biologically active compounds have been widely used for thousands years in prevention and treatment of many diseases including a variety of cancers, heart diseases, diabetes, skin diseases and so on [6]. Traditional Chinese medicine (TCM) has gained increased recognition internationally in recent years, but it has not been fully accepted by mainstream medicine because of lack of understanding of its complex nature of the formulae, as well as perceived lack of stringent quality control and regulations. ANBP is a pulverized mixture of four herbs, consisting of *Agrimonia Eupatoria* (A), *Nelumbo Nucifera Gaertn* (N), *Boswellia Carteri* (B) and *Pollen Typhae Angustifoliae* (P). ANBP is derived from an ancient formulation of herb medicine, which was first described in Chinese canonical medicine about 2000 years ago for treatment of various trauma disorders [7]. The active ingredients of herbal medicines are mainly composed of numerous of polysaccharides, saponins, flavonoids and amino acids. Recent studies indicate that A and P have multiple therapeutic properties including hemostasis, antiinflammatory, analgesia, wound healing and so on [8], [9].

Embryonic wounds appear to have an ideal tissue repair or healing process which leads to scar-free healing [10], [11]. Scar-free healing in embryonic tissue is believed to be related to the low levels of transforming growth factor (TGF)-β1 signaling in embryo compared to adults. TGF-β1 is one of the most powerful and widely distributed profibrogenic mediators in the body and regulates many of these processes [12]. Localized increase in the release and activation of TGF-β1 in burn injuries have delayed re-epithelialization and enhanced the scarring response [13]. TGF-β1 and TGF-β2 are known to promote scar tissue, while TGF-β3 may reduce scar formation [14], [15]. ANBP, consistently prepared by stringent manufacturing practices [16], [17], promoted wound healing and reduced hypertrophic scars in animal models and clinically. However, little is known about the precise pathological mechanisms of ANBP on wound healing. In this study, we further investigated the therapeutic effect of ANBP on the full-thickness excision in the rabbit ear models to explore the possible mechanisms. We demonstrated that ANBP not only promoted wound healing, but also reduced scar formation possibly via the bidirectional regulation of TGF-β1/Smad pathway.

## Materials and Methods

### Animals and Ethics Statement

New Zealand white rabbits (∼1.5 kg) were purchased from Beijing Vital River Laboratory Animal Technology Company. All animals were treated strictly in accordance with international ethical guidelines and the National Institutes of Health Guide concerning the Care and Use of Laboratory Animals. The rabbits were euthanatized with sodium intravenous injection of pentobarbital (30 mg/kg) (2^nd^ Pharmaceutical, Shanghai) after the experiment. The experiments were carried out with the approval of the Animal Experimentation Ethics Committee of Chinese PLA General Hospital.

### Preparation of Ultralow Temperature Broken ANBP Powder

The four herbs *Agrimonia Eupatoria* (A), *Nelumbo Nucifera Gaertn* (N), *Boswellia Carteri* (B) and *Pollen Typhae Angustifoliae* (P) (TongRenTang Pharmaceutical, Beijing) were authenticated by morphological characterization and thin layer chromatography in accordance with the *Chinese Pharmacopoeia*. The preparation method of ultralow temperature broken ANBP powder is in the duration of patent right (201210467065.8) protection. Briefly, ANBP were mixed by a certain ratio and titrated immediately in ultralow temperature to break the cell wall, and then filtrated by a 200-mesh sieve.

### Structural Properties of ANBP Powder

The ANBP powders were dehydrated using a freeze dryer. And then the powders were mounted on aluminum stubs, sputter-coated with a 5-nm-thick layer of platinum and observed with a scanning electron microscope (SEM, S-2500 Hitachi, Tokyo). Images were captured using the Quartz PCI system. The particle diameter of ANBP powder was obtained by static laser scattering (Analysette22, Fritsch, Idar-Oberstein). Ultraviolet liquid fingerprint analysis of ANBP powders was performed using high-performance liquid chromatography (API2000, Applied Biosystems, California) to control the quality.

### Model of wound healing

The rabbit ear model of hypertrophic scarring was established as described previously [18]. In brief, New Zealand white rabbits (n = 6, each group) were kept under standard conditions, anaesthetized with an intravenous injection of sodium pentobarbital (30 mg/kg) and prepared for wounding under sterile conditions. Four 10 mm dermal wounds of full-thickness circular defect were created on the ventral surface of each ear down to bare cartilage after removal of the perichondrium. The ANBP powder was applied to cover the wound areas of the experimental group immediately, while the control group received no treatment. The experiment was repeated in 3 times.

### Observation of wound healing and scar formation

Percentage wound closure at each time point was derived by the formula: [1− (current wound size/initial wound size)] ×100. The maximum protuberant heights of hypertrophic scars in rabbits were measured independently by two people using calipers, and the scar elevation index (SEI) — the ratio of total wound area tissue height to the area of normal tissue below the scar — was measured as previously described [19].

### Histological Analysis

Wounded skins were excised, fixed overnight in 4% buffered formalin solution and embedded in paraffin. Tissue sections (5 µm) were stained with hematoxylin and eosin (H&E) for morphological assessment. The collagen analysis of skin wounds were performed using Masson's Trichrome Stain Kit (Sigma) and Sirius red staining. Furthermore, the thin/thick ratio of epithelial thickness, the epithelial crawling distance and the thickness of the granulation tissue were assessed as quantitative indicators of wound healing and scarring. The average value was calculated from at least six different sections. All histological measurements were made independently by two observers blind to treatment assignments and averages of their results were captured.

### Immunohistochemical Staining

The immunohistochemical staining of TGF-β1 (Santa Cruz) was conducted. Sections were dewaxed and hydrated; endogenous peroxidase was blocked with 3% hydrogen peroxide for 10 min; nonspecific binding was blocked with 1% BSA for 30 min. Primary were applied for TGF-β1 (1∶150) overnight at 4°C. Biotinylated secondary antibodies were then applied at 1∶200 for 30 min, followed by incubation with horseradish peroxidase (HRP)-streptavidin at 1∶400 for 30 min. Color development was performed with DAB for 3 to 5 min for all samples, followed by haematoxylin counterstaining, dehydration and cover slipping. The immunopositive in fields was counted for per sections using Image-Pro Plus software (Nikon).

### Western Blot

Western blotting was performed as previously described [19]. Briefly, the scar tissues were lysed; then the protein samples were denatured and then separated on 10.6% polyacrylamide gels, and transferred to the polyvinylidene difluoride membrane. The membranes were incubated in TBS containing 5% nonfat milk and 0.05% Tween-20 for 1 h at room temperature and blotted with primary antibodies (dilution 1∶300) at 4°C overnight. The membranes were washed with TBST for 15 min the next day. Subsequently, the membranes were incubated with horseradish peroxidase-conjugated affinipure goat anti-rabbit antibody (dilution 1∶3000) for 1 h at room temperature and washed with TBST for 21 min. The membranes were then detected using ECL.

### Quantitative Real-Time Polymerase Chain Reaction (qRT-PCR)

Total RNA was isolated from all samples using TRIzol (Invitrogen) according to the manufacturer's instructions. RNA was quantified by UV spectrophotometer, and cDNA synthesis was conducted according to RNA PCR kit protocol (Invitrogen). The primer sequences are shown in Table 1. qRT-PCR amplification was performed in triplicate, using the SYBR Green I PCR reagent system (Applied Biosystem). During each extension step, SYBR green fluorescence was monitored and provided the real-time quantitative measurements of the fluorescence. Expression levels were calculated using standard curves and normalized to the expression level of β-actin as an endogenous reference gene.

**Table 1:** Primers used for real-time RT-PCR analysis.

Gene	Sense	Antisense
IL-1	TCCACTGTTTGTGAGTGCCC	GCCATGTGCACCAGATGTTC
TNF-α	GTAGTAGCAAACCCGCAAGTGG	CTGAAGAGAACCTGGGAGTAGATGAG
Col I	GTGTGGTTCGGCGAGAGCA	CAGTGGTAGGTGATGTTCTGGGA
Col III	GCCTCCCAGAACATCACCTATC	AGCAGCCATCCTCCAGAACT
TGF-β1	CTGAGAGGTGGAGAGGAAATAGAGG	CTGTGTAGATGTTGAGCCCGTTC
Smad2	AGGTTCTGCTTAGGGTTACTGTCC	GGTGCCAGCCGTATCTCTGAT
Smad3	GCTTCCAGACCGCACCTTCG	GCCGATCCCAGCAACACCAC
β-actin	CAACATGGTGCTGTCAGGCT	CCGCCGATTCACACCGAGTA

### Statistical Analysis

The data were expressed as the mean ± SEM. Statistical significance was determined with the student's t-test when there were two experimental groups. For more than two groups, statistical evaluation of the data was performed using one-way analysis of variance (ANOVA), followed by Dunnett's post-hoc test. *p*<0.05 or *p*<0.01 was considered statistically significant.

## Results

### Structural Properties of Ultralow Temperature Broken ANBP Powder

**Figure 1:**
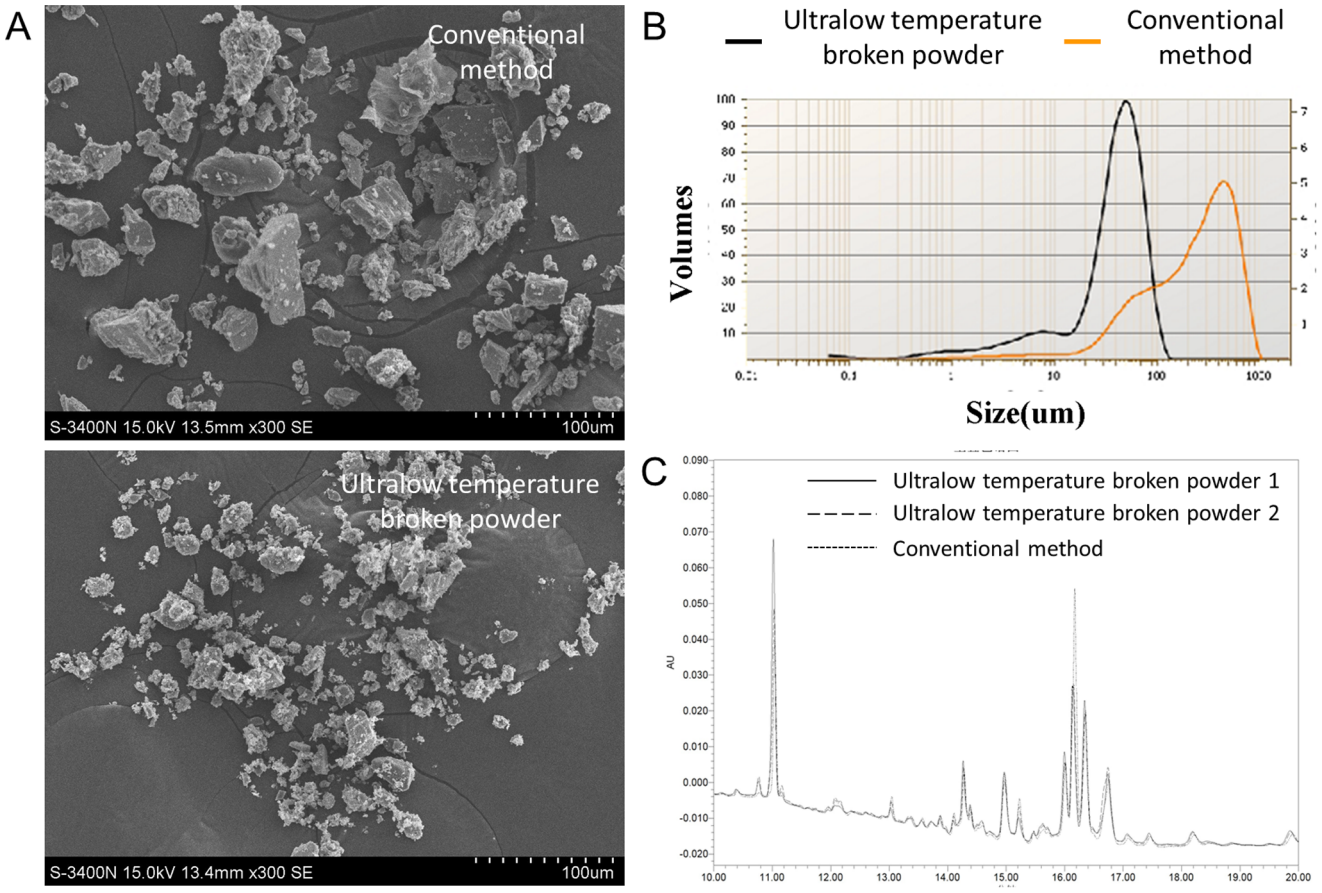
Structural properties of ultralow temperature broken ANBP powder. (A) Scanning electron microscope micrographs of ultralow temperature broken and conventional ANBP powders. (B) Static laser scattering analysis of dissolved ANBP particle size distribution(C) Liquid chromatography chromatograms of two different methods of ANBP powder preparation.

SEM micrographs of ultralow temperature broken ANBP powder showed uniform and micron-scaled granular structures, but the powder prepared by conventional method varied significantly in size. The diameters were40.58±1.35 µm and 74.08±26.03 µm for ultralow temperature broken and conventional ANBP powders, respectively (Fig. 1A). The swelling capacity showed similar profile of powders prepared by two different methods. Static laser scattering analysis showed that the average particle diameters of ultralow temperature broken and conventional, dissolved ANBP powders were significantly different. (Fig. 1B). The little difference of particle diameter was attributed to the different types of herb medicines. Two different methods of ANBP powder preparation exhibited similar profiles of liquid chromatography (Fig. 1C) and showed an excellent and consistent representation of the phytochemical components. These results indicated that ultralow temperature broken powder technology could provide an important basis for quality control of ANBP powder.

### ANBP Accelerates Wound Healing and Inhibits Scar Formation

**Figure 2:**
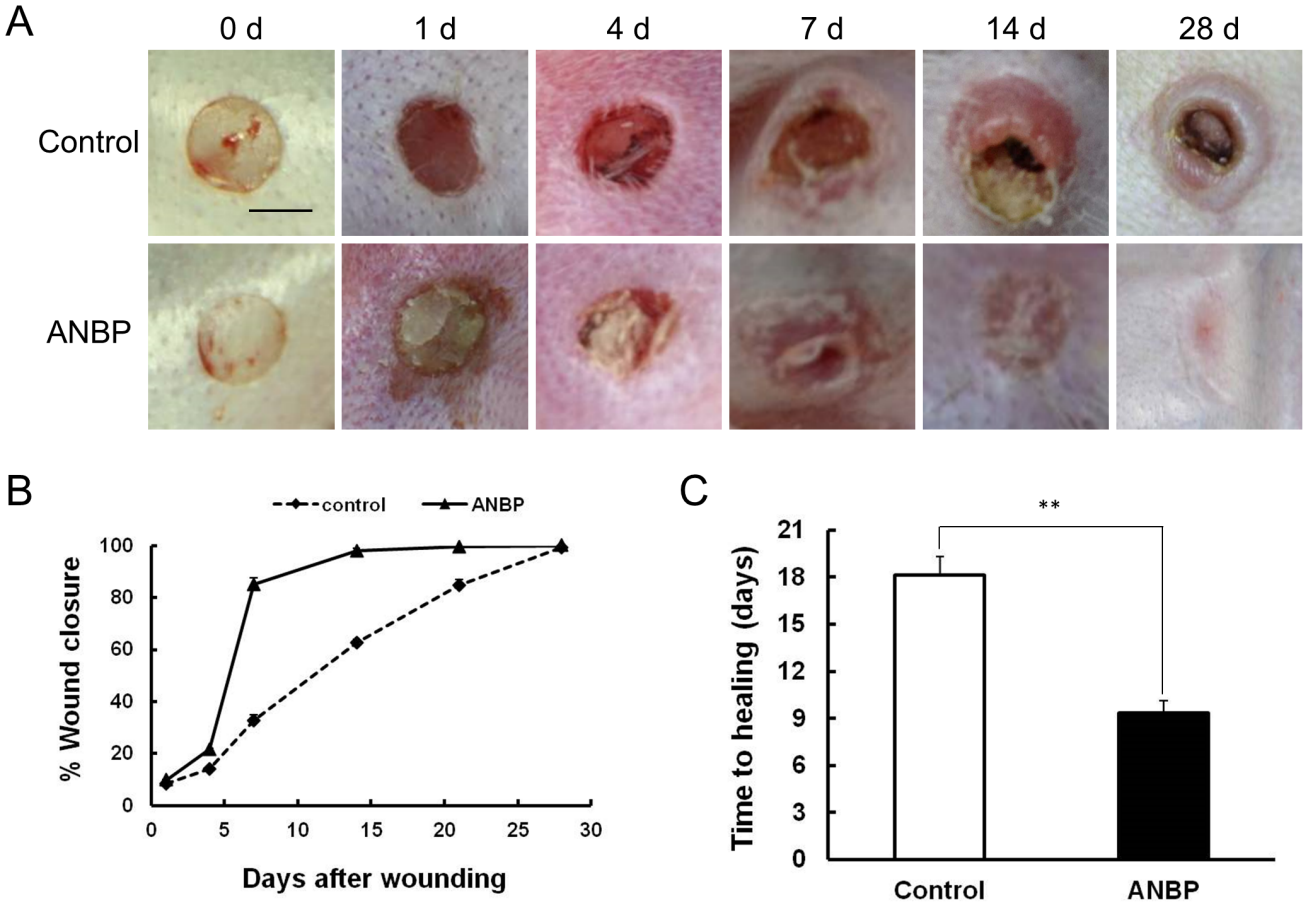
ANBP accelerates wound healing and inhibits scar formation. (A) Representative photographs of full-thickness skin wounds at various time points after treatment with or without ANBP powders. Scale Bar  = 5 mm. (B) Time course of wound closure for each experimental and control group (n = 6 for each group, repeated 3 times in total). (C) Comparing of wound healing time after treatment with or without ANBP powders. **p<0.01.

Comparison of wound size revealed clear enhancement of wound healing treated by ANBP compared to the control group, particularly at 7 days post-wounding (Fig. 2A). The wounds treated by ANBP recovered much more quickly with better skin appearance. The fastest healing in ANBP group occurred between 4 and 7 days post-wounding (Figure 2B), and ANBP treatment significantly shortened the healing time by about 50% compared to the control group (Fig. 2C). After day 14, the wounds treated with ANBP were almost scarless, while the wounds in the control had obvious scars (Fig. 2A).

### Histological Changes after ANBP Treatment

**Figure 3:**
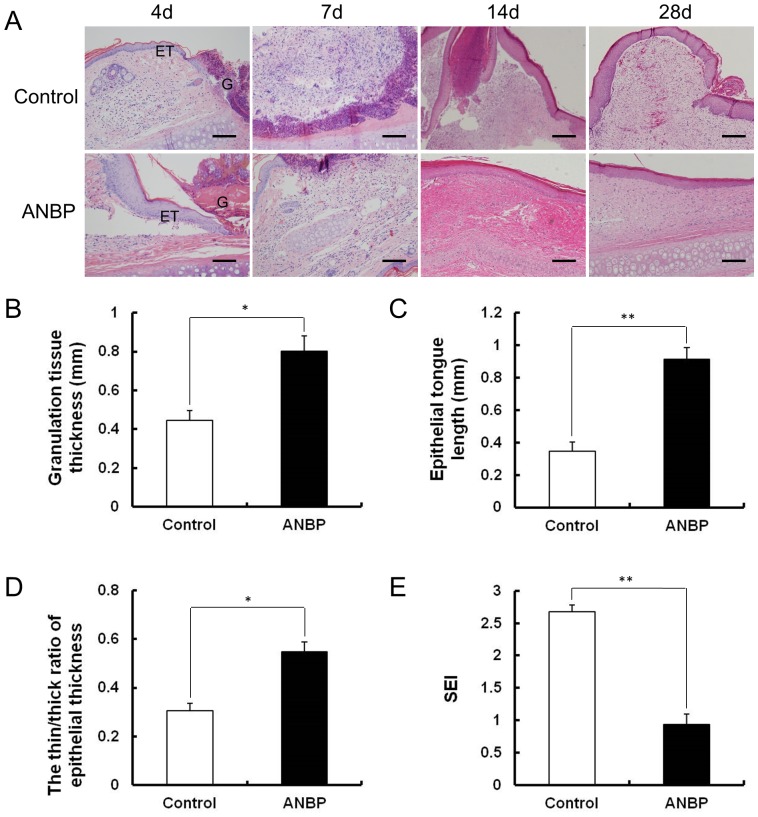
Histological characteristics of wound healing and scar formation after ANBP treatment. (A) Histological observation of wound healing at 4, 7, 14 and 28 days post-wounding with or without ANBP treatment. G: Granulation tissue, ET: Epithelial tongue. Scale bars  = 100 µm. (B) Quantitative analysis of granulation tissue thickness after 4 days post-wounding. * *p*<0.05. (C) Quantitative analysis of crawling distance after 4 days post-wounding. ** *p*<0.01. (D) The thin∶thick ratio of epithelial thickness at 28 days post-wounding. * *p*<0.05. (E) The scar elevation index (SEI) was significantly decreased on ANBP side at 28 days post-wounding. ** *p*<0.01.

These wound closure rates made a good match to the results of H&E staining (Fig. 3A). Compared with the control group, granulation tissue grew significantly faster after 4 days post-wounding for ANBP-treated wounds (Fig. 3B). In addition, re-epithelialization toward the centre of the wound was more rapid (Fig. 3C), and inflammatory cell infiltration decreased significantly on ANBP side. This difference was apparent between 7 and 14 days post-wounding. The epithelial thickness on ANBP group was similar to the surrounding normal skin. The thin∶thick ratio of epithelial thickness on the ANBP group was significantly higher than control (Fig. 3D). Quantitative analysis showed that the SEI was decreased by 60% on the ANBP group (Fig. 3E). Histologically, the dermis layer of the control scars thickened significantly, while the epidermis in the wounds treated with ANBP for 28 days was flattened and no hypertrophic scar appeared. These results demonstrated ANBP treatment accelerated wound healing and inhibited scar formation.

### Effect of ANBP on Extracellular Matrix Synthesis

**Figure 4:**
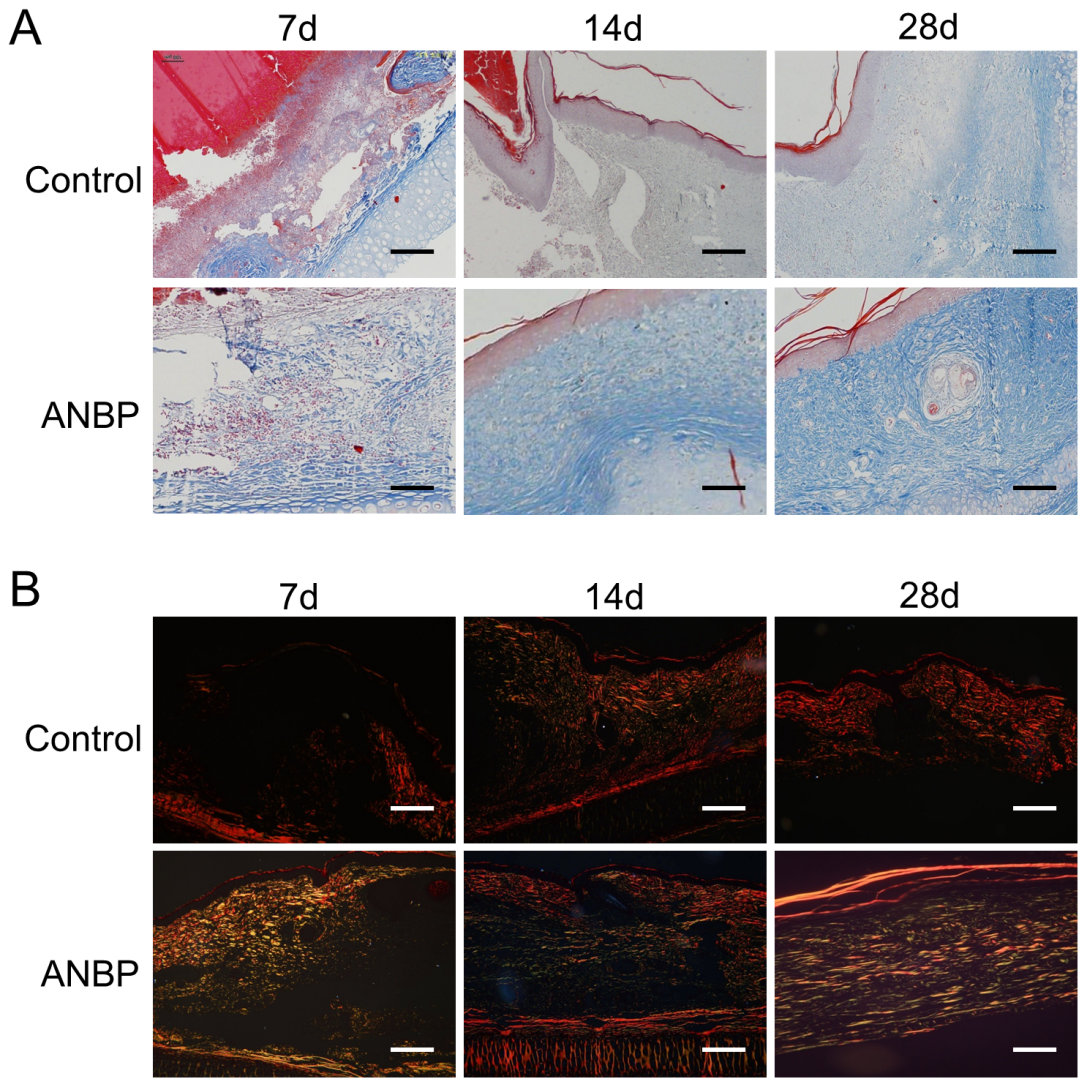
Effect of ANBP on extracellular matrix (ECM) synthesis. (A) Histopathological observation and masson staining of collagen in wound healing at 7, 14 and 28 days post-wounding with or without ANBP treatment. Scale bars  = 100 µm. (B) Sirius red staining of collagen in wound healing at 7, 14 and 28 days post-wounding with or without ANBP treatment. Scale bars  = 100 µm.

In order to evaluate the effects of ANBP on extracellular matrix (ECM) production, we conducted the masson staining of collagen which constituted the bulk of ECM. Although collagen staining was light and unevenly distributed at 7 days post-wounding, there was increased collagen content on the ANBP group compared to the control group (Fig. 4A). Collagen on the control side was still disorderly distributed and less mature at 14 days post-wounding, while at the ANBP side collagen was densely distributed, more mature and arranged parallel to the epithelium. Especially at 28 days post-wounding, control-side collagen fibres were evenly spaced and arranged in parallel with light coloration, while collagen on the ANBP side was darkly stained and arranged in a net-like shape similar to the adjacent unwounded tissue (Fig. 4A). To analyze the distribution of collagen type, wounded skin tissue sections were stained with Sirius red (Fig. 4B). Under the polarized light microscope, type I collagen and type III collagen showed red and yellow, respectively. The results of Sirius red staining showed that type III collagen accounted for the majority on the ANBP side during wound healing, but on the control side type I collagen accounted for the majority.

### Changes in Expression of Inflammatory Factors and Collagen after ANBP Treatment

**Figure 5:**
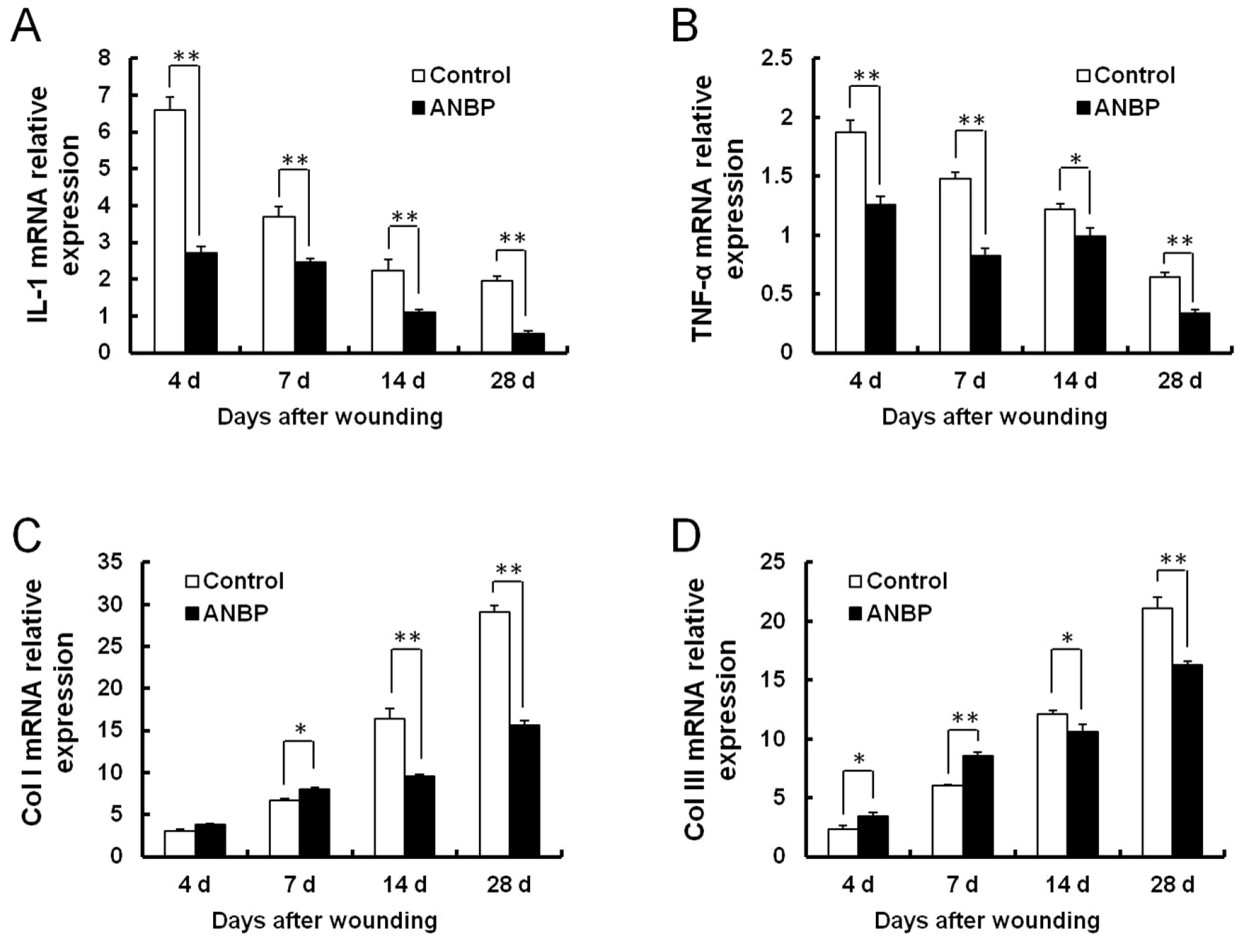
Changes in expression of inflammatory factors and collagen after ANBP treatment. mRNA levels were detected by quantitative RT-PCR at 4, 7, 14 and 28 days post-wounding. mRNA levels are shown for: (A) IL-1; (B) TNF-α; (C) Col I; (D) Col III. Expression levels were calculated using standard curves and normalized to expression level of β-actin as an endogenous reference gene. * *p*<0.05, ** *p*<0.01.

Inflammation is an important factor affecting the outcome of wound healing. Wounding induced large increases in IL-1 and TNF-α mRNA expression. Real-time PCR indicated that both were highest at the earliest time point examined (4 days post-wounding), declining thereafter over the time of healing (Fig. 5A and 5B). ANBP treatment significantly reduced the levels of inflammatory cytokines IL-1 and TNF-α at all time points. The mRNA levels of collagen I and collagen III increased further over time (Fig. 5C and 5D). Compared with the control, ANBP treatment slightly increased collagen I and collagen III synthesis during the early post-wounding period (7 days). But at the later time of healing (14 days), expressions of collagen I and collagen III were both reduced by ANBP treatment.

### Effect of ANBP on TGF-β1/Smad Pathway

**Figure 6:**
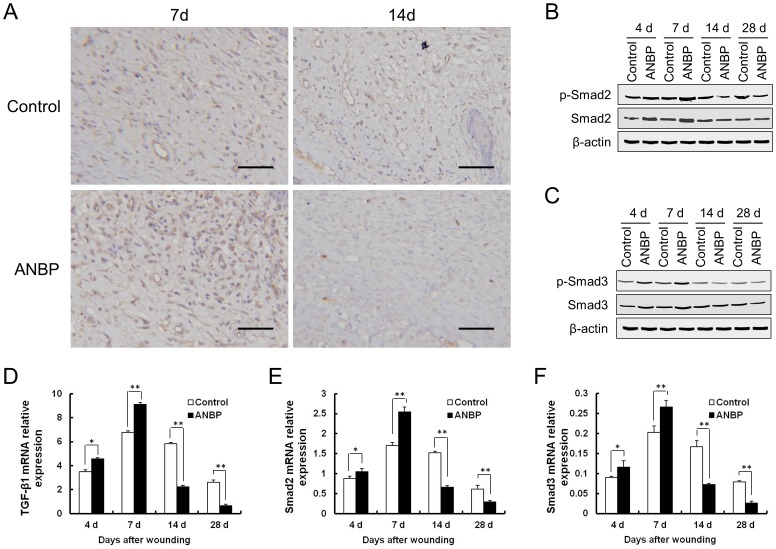
Effect of ANBP on TGF-β1/Smad Pathway. (A) Immunohistochemistry for TGF-β1 in the wound granulation tissue at 7 and 14 days post-wounding. Scale bar  = 50 µm. Expressions of Smad2 (B) and Smad3 (C) were detected by western blot at 4, 7, 14 and 28 days post-wounding. mRNA levels were detected by quantitative RT-PCR at 4, 7, 14 and 28 days post-wounding. mRNA levels are shown for: (D) TGF-β1; (E) Smad2; (F) Smad3. * *p*<0.05, ** *p*<0.01.

Compared with the control group, expression amounts of TGF-β1 in the ANBP group significantly increased on day 7. On the 14th day, the expression amounts of TGF-β1 levels on ANBP side were significantly decreased compared to the control (Fig. 6A). Real-time PCR indicated that TGF-β1 expression levels began to increase after injury and peaked at 7 days, markedly decreasing at 14 days post-wounding (Fig. 6D). Similarly, the protein and mRNA levels of Smad2 and Smad3 increased significantly by 7 days post-wounding, and then decreased gradually on day 14 and (Fig. 6B, 6C, 6E, and 6F). ANBP treatment significantly increased expression of TGF-β1 mRNA, particularly 4–7 days post-wounding. At the same time, the levels of phosphorylated Smad2 (p-Smad2) and Smad3 (p-Smad3) increased during 4–7 days post-wounding after ANBP treatment. Unexpectedly, at the later time of healing, ANBP treatment led to markedly decrease expression of TGF-β1, p-Smad2 and p-Smad3 compared with the control group after 14 days post-wounding (Fig. 6B and 6C).

## Discussion

Skin wound healing is a cascade of regulated biomolecular events for regeneration and restoration of the tissue. There is a contradiction inherent in trying to treat wound healing and scar formation simultaneously, because scar formation could be seen as the result of over-active wound healing. Herb medicine has been practiced for almost all of diseases, including wound healing more than 2,000 years in China. Here, we explored the effect of four-herb Chinese medicine ANBP on wound healing and scar formation in rabbit ear hypertrophic scar models of full-thickness skin defect. Compared with the control group, local ANBP treatment not only significantly enhanced wound healing, but also reduced scar formation.

The electron micrographs and laser light scattering images of ultralow temperature broken ANBP powder revealed that the particle diameter was uniform (Chinese patent right, 201210467065.8). The ultralow temperature broken ANBP powder has a well swelling property and bioavailability, making it easy to improve drug activity. When used externally for emergency wound, ANBP powder mixed with body fluid and blood could swell and cover the wound. More uniform the ANBP particles are, better the therapeutic effect is. So, the ultralow temperature broken powder method was significantly better than the conventional method. Three different batches of ANBP powder exhibited similar profiles of liquid chromatography. The results indicated that ultralow temperature broken powder method can provide a stable system of quality control standard authentication for clinical applications, but need further study for bulk drug production.

Wound repair is divided into three phases: inflammatory response, proliferation stage, and scar formation [20]. Inflammation is an important factor affecting the outcome of wound healing [21], [22]. We found that ANBP treatment not only reduced inflammatory cell infiltration in the wound but also significantly reduced levels of the inflammatory cytokines IL-1α and TNF-α, suggesting that ANBP treatment could reduce the inflammatory response during wound healing. In addition, ANBP treatment significantly induced rapid closure of the wound and increased the formation of granulation tissue at 7 days post-wounding. Furthermore, re-epithelialization was significantly accelerated following ANBP treatment. Inhibiting inflammation, speeding up granulation formation and accelerating re-epithelialization have all been reported to aid the wound healing process [23]–[26], and ANBP treatment may promote wound healing via all three mechanisms of action.

Fetal scarless healing hinted that the distribution and type of collagen in the process of fetal wound healing are different from the adults, leading to completely different healing outcome [27]. In the fetal scarless healing, collagen was mainly type III collagen, sparsely distributed and arranged in a net-like shape. However, the collagen was mainly type I collagen, densely distributed and arranged parallel in adult skin healing. We found that ANBP could accelerate wound healing with increasing collagen production during the early wound healing process and inhibit scar formation with subsequent collagen deposition. The outcome is an improvement in the quality of wound healing. ANBP treatment resulted in a sparse arrangement of the collagen, similar to normal skin collagen distribution, reduced collagen content and prevented the formation of the nodular structures typically found in scar tissue. Interestingly, the ratio of type III collagen/type I collagen in the later stages of wound healing was significantly higher in ANBP group compared to the control. These results demonstrated that ANBP treatment not only reduced collagen synthesis and blocked excessive deposition of collagen but also promoted collagen maturity, thus inhibiting the formation of scar. The mechanism of the effect of ANBP on collagen expression is different in the early and late stages of wound healing, which is beneficial for wound closure and scar diminution.

Smad2/Smad3 signalling system of the TGF-β1/Smad-dependent pathway is considered an important pathway in scar formation [28]–[30]. Our hypothesis was that ANBP treatment may promote wound healing and reduce scarring by regulating the TGF-β1/Smad-dependent pathway. These outcomes cannot be achieved simultaneously by inhibiting either TGFβ1 or Smad proteins alone. Here, we found that ANBP treatment significantly increased the protein and mRNA expression of TGF-β1, Smad2 and Smad 3 during the early stage post-wounding, while the expressions of TGF-β1, Smad2, Smad3 were markedly decreased on the ANBP group at the later time of healing. These results supported the notion that ANBP may act by regulating TGFβ1/Smad-dependent pathway. ANBP treatment could promote wound healing early due to activation of the TGF-β1 signaling pathway, and inhibit scar formation at later stages due to inhibition of the TGF-β1 signaling pathway. Taken together, these findings suggest that ANBP promote wound healing and reduce scarring by bidirectional regulation of the TGF-β1/Smad-dependent pathway.

In summary, we provide evidence of a new therapeutic strategy that ANBP plays dual roles by both promoting wound healing and alleviating scar formation in rabbit ear models. Our data indicate that the potential therapeutic effects of ANBP are better than those of existing measures to promote wound healing and inhibit scar formation, strongly suggesting that it is possible to achieve these seemingly contradictory effects with a single treatment. Ultralow temperature broken ANBP powder holds promise for future clinical applications to treat non-healing or abnormal wounds and hypertrophic scars.
